# Anterior Scalene Muscle Block for Diagnostic and Surgical Planning in Pediatric Thoracic Outlet Syndrome—Two Case Reports

**DOI:** 10.3390/children12070873

**Published:** 2025-07-02

**Authors:** Dahye Park, Mihaela Visoiu

**Affiliations:** 1 Department of Anesthesiology and Perioperative Medicine, UPMC Presbyterian, University of Pittsburgh Medical Center, Pittsburgh, PA 15213, USA; parkd6@upmc.edu; 2 Department of Anesthesiology and Perioperative Medicine, UPMC Children’s Hospital of Pittsburgh, University of Pittsburgh School of Medicine, Pittsburgh, PA 15224, USA

**Keywords:** thoracic outlet syndrome, anterior scalene muscle block, regional anesthesia, adolescent, acute pain

## Abstract

**Background/Objectives**: Thoracic outlet syndrome (TOS) is a rare and difficult-to-diagnose condition in children, characterized by the compression of neurovascular structures in the thoracic outlet. Neurogenic TOS (nTOS) often presents with nonspecific symptoms such as paresthesia, weakness, and upper extremity discomfort. While anterior scalene muscle block (ASMB) has been used in adults as a diagnostic adjunct, its role in pediatric patients remains underreported. **Methods:** We present two adolescent female patients with suspected neurogenic thoracic outlet syndrome (nTOS) who were referred to the acute pain service for further evaluation. Both patients underwent ultrasound-guided ASMB. **Results**: Following the block, both patients experienced rapid and marked relief of symptoms. Subsequently, each underwent first rib resection with brachial plexus neurolysis. At follow-up, both patients reported a complete resolution of symptoms and a return to baseline function. **Conclusions**: These cases suggest that ASMB may serve as a functional diagnostic tool and short-term therapeutic test in pediatric nTOS patients. It also helps guide surgical decision-making for invasive treatment. However, as ASMB is not without risk, its role should be considered supportive rather than confirmatory. Further studies are needed to better define its utility and safety in the pediatric population.

## 1. Introduction

Thoracic outlet syndrome (TOS) is an uncommon and challenging condition to diagnose. It includes a spectrum of conditions caused by the compression of neurovascular structures within the thoracic outlet, a space bordered by the clavicle, first rib, and scalene muscles [1]. The commonly associated symptoms include pain, fatigue, pallor, cyanosis, coldness of the limb, muscle heaviness and weakness, paresthesia, and motor deficits [2].

Thoracic outlet syndrome (TOS) is classified into three subtypes based on the anatomical structure involved: neurogenic TOS (nTOS), resulting from brachial plexus compression; venous TOS (vTOS), due to subclavian vein compression; and arterial TOS (aTOS), involving compression of the subclavian artery [1]. Neurogenic thoracic outlet syndrome (nTOS) is defined as upper extremity symptoms extending beyond the distribution of a single cervical nerve root or peripheral nerve, persisting for at least 12 weeks, not satisfactorily explained by other conditions, and meeting at least one criterion in at least four of the following five diagnostic categories. These categories include principal symptoms such as pain in the neck, upper back, shoulder, arm, and/or hand; numbness, paresthesia, and/or weakness in the arm, hand, or digits; and clinical history, physical examination findings, and results from provocative maneuvers [3].

TOS in pediatric populations is relatively rare compared with that in adults and often presents with vague, nonspecific symptoms, such as neck or shoulder pain and upper extremity discomfort, leading to frequent misdiagnoses or delays in diagnosis [2,4]. Traditional diagnostic approaches include a thorough medical history, clinical presentation, physical examination such as provocation tests, and imaging modalities like magnetic resonance imaging (MRI). Electrophysiological studies may also be used to rule out other entrapment syndromes. However, these diagnostic tools often fail to provide conclusive evidence due to the high false-positive rate of provocative tests, limited cooperation from younger patients, and the dynamic nature of TOS [1]. As a result, the diagnosis of pediatric TOS is frequently delayed—ranging from 2 to 60 months—often involving extensive imaging and consultations with multiple specialists [5]. Treatment for TOS primarily included muscle resection, followed by neurolysis and bone resection, with frequent overlap in procedures that included two or more of these interventions. Patients with early-stage TOS can be treated with conservative management, such as rest and posture correction [6]. However, it is difficult to diagnose early-stage TOS. Surgical intervention is regarded as a definitive treatment targeting the underlying cause of thoracic outlet syndrome. In pediatric patients, early decompression is particularly important due to ongoing growth and development [6], which may exacerbate symptoms or lead to long-term functional impairment if left untreated.

Anterior scalene muscle block (ASMB) offers a therapeutic test with diagnostic value, particularly in neurogenic cases. It temporarily relieves neurovascular compression through targeted muscle relaxation and may assist in the diagnostic evaluation of neurogenic TOS (nTOS), potentially supporting the decision to proceed with surgical intervention [1,7,8,9,10,11].

In this case report, we demonstrate the diagnostic and short-term therapeutic utility of ASMB in guiding surgical decision-making for pediatric patients with suspected neurogenic TOS, addressing a gap in the current literature.

Informed consent for participation was obtained from the parents or guardians of all patients involved in the study.

## 2. Case Descriptions

### 2.1. Case 1

A 12-year-old girl weighing 48.9 kg with a remote history of migraines with aura presented with a 9-month history of progressively worsening neurologic symptoms in her right upper extremity. Before consultation with the acute pain service for diagnostic ASMB, the patient had been evaluated by multiple specialists and underwent extensive diagnostic testing and conservative treatment. However, none of the interventions provided definitive symptom relief. She was subsequently referred to our service to assist in confirming a diagnosis of TOS using ASMB. Her symptoms began suddenly following intense exercise and initially manifested as numbness, tingling, weakness, and swelling in the wrist, hand, and proximal forearm, later spreading to the entire arm. She also reported worsened tingling in her right upper extremity when her shoulders were elevated. She experienced daily flare-ups, along with hand discoloration. Over time, the symptoms intensified, with an increasing frequency of edema and a purplish discoloration of the hand. Although she reported no pain, functional limitations prompted her to switch from right- to left-handed for tasks such as handwriting. The patient reported headaches with photophobia and occasional phonophobia, which she described as subjectively different from her prior history of migraines with aura. On physical examination, the peripheral pulses were intact, with no evidence of vascular compromise. The Elevated Arm Stress Test (EAST) reproduced tingling sensations in the right fingers, suggesting neurogenic involvement. In contrast, the Adson test (used to assess nTOS) and the Wright test (to evaluate vTOS) were unremarkable. Neurologic examination revealed right-hand weakness (4/5), numbness throughout the arm, and mild swelling in the forearm and hand. Electromyography (EMG) and nerve conduction studies were performed twice, yielding normal results. Chest magnetic resonance angiography (MRA) demonstrated positional compression of the subclavian vein without evidence of thrombosis, raising concern for nTOS.

**Figure 1:**
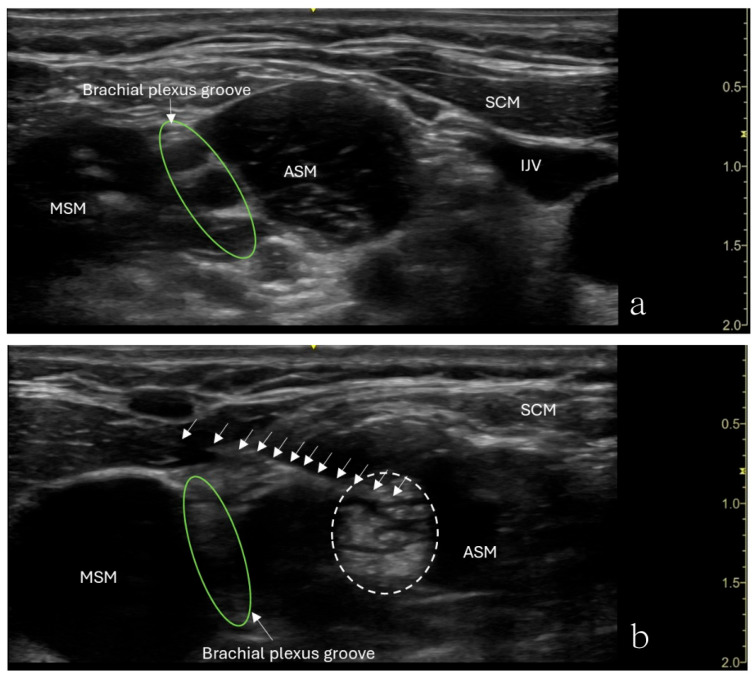
Ultrasound image showing an anterior scalene muscle block in a 12-year-old girl. (**a**) SCM—sternocleidomastoid muscle, MSM—middle scalene muscle, ASM—anterior scalene muscle, IJV—internal jugular vein. The interscalene nerve roots (C5,6,7) are highlighted with a green circle. (**b**) The needle (arrows) is inserted into the middle of the anterior scalene muscle (ASM) from the posterior aspect of the neck anteriorly, with local anesthetic spreading within the muscle (dashed line). The brachial plexus groove lies between the ASM and MSM.

The patient underwent an ultrasound-guided right anterior scalene muscle block under minimal sedation. A high-frequency linear probe was used to identify the anterior scalene muscle (Figure 1). After sterile preparation, a 22-gauge, 50 mm Sono-TAP needle (PAJUNK^®^, Geisingen, Baden-Württemberg, Germany), using an in-plane technique, was advanced from the posterior aspect of the neck anteriorly and was inserted into the middle of the anterior scalene muscle. After negative aspiration of blood, 5 mL of 0.5% ropivacaine was slowly injected in increments. An increased muscle size was observed with no medication extravasation to the brachial plexus divisions (Figure 1). After the procedure, the patient’s preexisting numbness disappeared, her weakness in her right shoulder disappeared, and her hand strength improved. However, she reported shoulder heaviness though she had a full range of motion.

Following the ASMB procedure, the patient was considered a candidate for surgery, and a few months later, she underwent first rib excision and brachial plexus neurolysis. At the one-month postoperative follow-up, the patient’s symptoms had entirely resolved. The use of ASMB increased the likelihood of a TOS diagnosis, leading to direct referral to the TOS clinic. Although the patient had to wait a few months for the invasive procedure due to age considerations, symptoms were effectively managed with botulinum toxin injections into the anterior scalene muscle under the diagnosis of nTOS. This approach allowed the patient to eventually undergo the surgical procedure.

### 2.2. Case 2

A 15-year-old girl, weighing 54.8 kg, developed progressively worsening right upper extremity pain (8–9/10) over 9 months. Additional symptoms included sharp, radiating pain from the right shoulder to the fingers, paresthesia, decreased sensation, and daily headaches from the neck to the orbital region. One of the distinctive symptoms was paresthesia, which worsened with right arm abduction. She also reported symptom exacerbation after starting volleyball—an activity involving frequent overhead movements of the dominant arm.

The patient initially presented to orthopedic specialists to evaluate for bone-related abnormalities. Cervical spine MRI revealed minimal central disc protrusions at the C4–5 and C5–6 levels without evidence of a spinal canal or foraminal narrowing. Patients with congenital anomalies involving bony and muscular structures were also excluded. Following multiple sessions of physical therapy and conservative management without clinical improvement, the patient was referred to neurology. The brain MRI findings were unremarkable; however, concern for brachial plexus pathology emerged. Subsequent dynamic MRI of the brachial plexus demonstrated right subclavian vein compression within the costoclavicular space during arm abduction, suggestive of combined venous and neurogenic thoracic outlet syndrome (vTOS and nTOS). Given the clinical presentation and imaging findings, the surgical team sought to assess the patient’s symptom responsiveness through a functional diagnostic approach prior to considering definitive surgical intervention. As a result, the patient was referred to the acute pain service for an ASMB.

**Figure 2:**
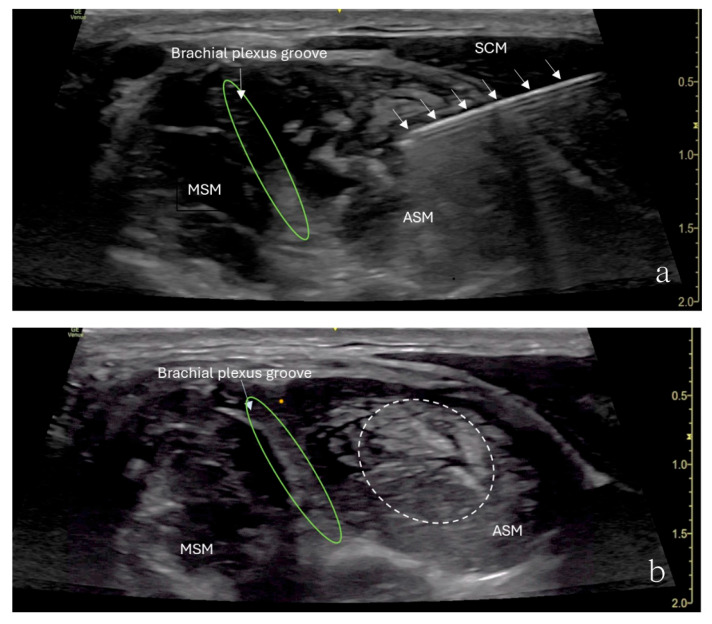
Ultrasound image showing an anterior scalene muscle block in a 15-year-old girl. (**a**) Needle (arrows) inserted into the anterior scalene muscle (ASM), with the needle tip placed in the middle of the anterior scalene muscle. The needle was advanced from the anterior aspect of the neck posteriorly. (**b**) Brachial plexus groove (green circle) is in between the ASM and MSM. Injected local anesthetics (dashed circle) and an increased muscle size were noticed.

The patient underwent ultrasound-guided ASMB under minimal sedation. A high-frequency linear ultrasound probe was used to identify the anterior scalene muscle and adjacent neurovascular structures. After sterile preparation, a 24-gauge, 40 mm Sono-TAP needle (PAJUNK^®^, Geisingen, Baden-Württemberg, Germany), using an in-plane approach, was advanced from the anterior aspect of the neck posteriorly and inserted into the middle of the anterior scalene muscle (Figure 2). After negative aspiration, 5 mL of 0.5% ropivacaine was injected incrementally (Figure 2). The block provided immediate pain relief, and there was no numbness in the right upper extremity when the arm was elevated or abducted. However, the patient developed right-sided Horner’s syndrome and experienced weakness in the right shoulder, both of which were resolved shortly after the procedure. Her preexisting symptoms were resolved for approximately 3 h following the block.

Two months later, the patient underwent a first rib excision. Postoperatively, the patient reported a significant reduction in pain and complete resolution of paresthesia in the arm and hand. Symptom relief following ASMB led to direct referral back to the TOS clinic, where the surgeon decided to proceed with the invasive surgical procedure. Both the patient and the patient’s parents expressed satisfaction with ASMB as a diagnostic tool and its role in guiding further treatment.

## 3. Discussion

Initially introduced in 1934 for scalenus anticus syndrome, ASMB is now a valuable diagnostic tool for TOS [9,12]. This technique involves injecting a local anesthetic into the anterior scalene muscle to relax it, temporarily reducing pressure on neurovascular structures [7]. If symptoms improve, this indicates that compression in this region contributes to TOS, supporting the presumed diagnosis [8].

Several studies have demonstrated the diagnostic value of anterior sealene muscle block (ASMB) for TOS. Braun et al. reported improved work performance in suspected TOS patients [8], Beason et al. identified correlations with postoperative outcomes [9], and Rached et al. observed pain and functional improvements following ultrasound-guided ropivacaine injections [11]. While ASMB is well established in adult TOS diagnosis, its role in pediatric patients is underexplored, with no large studies on pediatric TOS available. In pediatric TOS diagnosis, studies have relied primarily on clinical assessment and imaging techniques like MRI, duplex ultrasonography, and electrodiagnostic studies [2,4,5,13,14]. However, none of these studies included ASMB or other nerve blocks as part of the diagnostic process.

These cases highlight its clinical utility in adolescents, where TOS diagnosis is challenging due to its rarity, nonspecific symptoms, and overlap with other musculoskeletal and neurological conditions. ASMB provides immediate symptom relief, supports the presence of neurovascular compression, and may facilitate timely diagnosis. This approach may reduce the need for extensive imaging studies and specialist consultations, allowing for earlier intervention.

ASMB requires the needle to be inserted into the anterior scalene muscle to avoid the risk of inadvertent brachial plexus block. In the first patient, the needle direction from the back towards the front led to shoulder weakness. We suspected local anesthetic backflow along the needle track during the injection and traveled toward the trunk of the brachial plexus. To avoid this recurring, the direction from the front towards the back was adopted for the injection for the second patient, which was very gradually delivered to ensure that the medication was being administered in the muscle. Unfortunately, the second patient showed temporary signs suggestive of a partial block of the brachial plexus and Horner’s syndrome. When given into the scalene muscle, the anesthetic may diffuse toward the trunk of the brachial plexus at the level of the triangle of the scalene muscles or block the trunk of the brachial plexus from passing through the anterior scalene muscle [6]. Less risk for a partial block of the brachial plexus can be achieved using a smaller volume of local anesthetic, although pediatric-specific guidelines for anterior scalene muscle block (ASMB) are currently unavailable. To date, no published data exist on local anesthetic dosing for ASMB in the pediatric population. However, several studies in adults have described the use of small volumes of local anesthetic for this purpose. According to a literature review, unilateral ASMB using 3 mL of 1% lidocaine was performed in a cohort of 34 patients (mean age: 46 years) [8]. Another study reported the injection of 3 mL of 1% lidocaine into the anterior scalene muscle belly (mean age: 47.8 years) [9]. Additionally, one study described the use of 1 mL of 1% lidocaine subcutaneously and 2 mL of 0.5% bupivacaine (mean age: 42 years) [10], while another reported using 4–5 mL of 1% lidocaine in adult patients [15]. In the absence of pediatric-specific dosing guidelines for ASMB, we referred to pediatric safety recommendations for peripheral nerve blocks of the upper extremity. These findings suggest a dosing range of 0.1–1.5 mg/kg for bupivacaine, levobupivacaine, or ropivacaine [16].

In our study, we used 5 mL of 0.5% ropivacaine (5 mg/mL), totaling 25 mg. Given the patients’ body weights (48.9 kg and 54.8 kg), the administered dose remained within the recommended safe range. It is also worth noting that the ASMB is a muscle plane injection rather than a traditional nerve block, and as such, we intentionally used a conservative dose. An anterior scalene muscle block (ASMB) is not a fascial plane block but rather a targeted intramuscular injection. Therefore, a higher concentration, such as 0.5% ropivacaine, is preferred over lower concentrations to ensure adequate muscle relaxation. Ropivacaine is also considered a safer option than bupivacaine in pediatric patients due to its reduced cardiotoxicity. Additionally, unlike lidocaine, which is short-acting, ropivacaine offers prolonged duration, providing both diagnostic and therapeutic benefits from a single injection.

In both cases, patients underwent surgical intervention after experiencing significant symptom relief with ASMB. These favorable outcomes support the utility of ASMB as a diagnostic adjunct and a tool for guiding surgical decision-making in TOS.

This report is not without limitations. ASMB provides only temporary symptom relief and is not a definitive test for TOS; it should be interpreted alongside other diagnostic methods. Additionally, most ASMB research focuses on adults with limited pediatric data.

In conclusion, these case reports demonstrate ASMB’s value as a functional diagnostic tool with short-term therapeutic value for pediatric nTOS, offering immediate symptom relief and confirming neurovascular compression. ASMB helps guide treatment decisions, including surgery, with temporary and self-limiting side effects. While effective, further studies are necessary to refine its role in pediatric care and optimize its clinical application.

## Data Availability

The data presented in this study are available on request from the corresponding author due to privacy reasons.
